# Opportunities for concurrent transcranial magnetic stimulation and electroencephalography to characterize cortical activity in stroke

**DOI:** 10.3389/fnhum.2015.00250

**Published:** 2015-05-05

**Authors:** Sumire Sato, Til Ole Bergmann, Michael R. Borich

**Affiliations:** ^1^Division of Physical Therapy, Department of Rehabilitation Medicine, Emory University School of MedicineAtlanta, GA, USA; ^2^Institute of Psychology, Christian-Albrechts University KielKiel, Germany

**Keywords:** stroke, transcranial magnetic stimulation, electroencephalography, TMS-EEG, rehabilitation, cortical excitability, connectivity

## Abstract

Stroke is the leading cause of disability in the United States. Despite the high incidence and mortality of stroke, sensitive and specific brain-based biomarkers predicting persisting disabilities are lacking. Both neuroimaging techniques like electroencephalography (EEG) and non-invasive brain stimulation (NIBS) techniques such as transcranial magnetic stimulation (TMS) have proven useful in predicting prognosis, recovery trajectories and response to rehabilitation in individuals with stroke. We propose, however, that additional synergetic effects can be achieved by simultaneously combining both approaches. Combined TMS-EEG is able to activate discrete cortical regions and directly assess local cortical reactivity and effective connectivity within the network independent of the integrity of descending fiber pathways and also outside the motor system. Studying cortical reactivity and connectivity in patients with stroke TMS-EEG may identify salient neural mechanisms underlying motor disabilities and lead to novel biomarkers of stroke pathophysiology which can then be used to assess, monitor, and refine rehabilitation approaches for individuals with significant disability to improve outcomes and quality of life after stroke.

## Introduction

Stroke is the fourth leading cause of death (Kochanek et al., 2011) and the leading cause of long-term disability in the United States (Go et al., 2014). Stroke risk increases with age and the population aged ≥65 years is expected to grow significantly in the next two decades (Ovbiagele et al., 2013). From 2012 to 2030, direct medical costs are projected to increase 250%; the majority of this increase will be for patients 65–79 years (Ovbiagele et al., 2013).

Substantial progress has been made in the identification and treatment of stroke-related disability, but the majority of evidence is based on heterogeneous studies that cannot be used to support robust conclusions regarding optimal choice of intervention on a patient-by-patient basis (Pollock et al., 2014). There is no consensus on the best rehabilitation approach and it remains unclear which patients will benefit most from a given intervention. There is a critical need to identify, validate and implement accurate biomarkers associated with underlying persisting disability in patients recovering from stroke.

Evidence from animal models and humans suggests there are widespread changes in cortical network activity patterns remote to the lesion that extend into the contralesional hemisphere during stroke recovery (Nelles et al., 1999; Takatsuru et al., 2009; Mohajerani et al., 2011). Functional impairments following stroke are a result of direct ischemic loss of neurons combined with maladaptive brain reorganization (Taub et al., 1994). Stroke triggers alterations in neuronal excitability with structural and functional reorganization of brain networks (Murphy and Corbett, 2009; Carrera and Tononi, 2014). Reorganization and remodeling of network connections contributes to recovery of motor function after stroke (Grefkes et al., 2010).

Neuroimaging and non-invasive brain stimulation (NIBS) have shown promise in identifying neurophysiological mechanisms associated with network reorganization in the human brain after stroke. Neuroimaging approaches have identified functional neural correlates of impaired arm movements after stroke demonstrating relationships between brain function and motor impairment (Platz et al., 2000; Carey et al., 2002; Gerloff et al., 2006). NIBS has been used to both characterize and modulate cortical activity in patients with stroke (for review Liew et al., 2014). Despite the important insights gained regarding stroke pathophysiology, a comprehensive characterization of the principal neural substrates mediating recovery of motor function after stroke remains elusive and is a barrier to optimizing rehabilitation strategies.

Combinations of neuroimaging and NIBS techniques have emerged in an attempt to better understand the complex spatiotemporal neural network dynamics comprising normal brain activity and abnormal activity in clinical populations (Sporns et al., 2004; Siebner et al., 2009). Of particular interest is combining electroencephalography (EEG) recordings during transcranial magnetic stimulation (TMS). Combined TMS-EEG offers a powerful “perturb and measure” paradigm to study induced neural activity in local and distributed brain networks. To date, TMS-EEG has yielded important insights into the pathophysiology of neural disorders and disease (Julkunen et al., 2011; Barr et al., 2013; Ragazzoni et al., 2013) but to our knowledge no studies have yet been performed with patients after stroke.

We first briefly summarize pertinent research contributions utilizing EEG and TMS in isolation to the current understanding of the neural mechanisms underlying functional recovery after stroke. We then discuss opportunities for TMS-EEG to identify new brain-based biomarkers of disability and highlight future clinical applications in chronic stroke.

## EEG

EEG is a low cost, noninvasive functional neuroimaging technique utilizing electrodes located on the scalp to quantify neuroelectric potentials in the brain. These potentials arise from a mixture of inhibitory and excitatory postsynaptic potentials in neurons that can be captured at the scalp and analyzed to assist in the diagnosis, prognosis, and monitoring of treatment response of a variety of different neurologic disorders (Olejniczak, 2006). EEG can detect specific cortical activation patterns in response to a given event or stimulus. Event-related potentials (ERPs) due to a peripheral somatosensory stimulus (SEPs) are partially generated in contralateral somatosensory processing regions. In acute stroke, abnormal SEPs have been correlated with increased length of inpatient rehabilitation stay and poorer functional outcomes (Feys et al., 2000).

Spontaneous synchronization of large neuron populations detected using EEG provides a non-invasive, systems-level surrogate marker of intrinsic neuronal network activity. Ongoing synchronized activity in M1 generates oscillations in the beta frequency range (15–30 Hz) at rest and during isotonic contraction (Baker et al., 1997). During volitional hand movement, beta oscillations desynchronize and thus decrease in amplitude (Pfurtscheller and Berghold, 1989). These oscillations appear to be controlled by gamma-aminobutyric acid (GABA) activity (Jensen et al., 2005; Hall et al., 2010). Disruptions in movement-related modulations in beta activity have recently been shown in chronic stroke, suggesting abnormal GABAergic activity (Rossiter et al., 2014). Quantitative EEG using power spectrum analysis of frequency band content has been applied in stroke showing correlations between spectral power and disability level after stroke (for review: Finnigan and van Putten, 2013).

Despite the clinical advantages, EEG has limitations, primarily low spatial resolution. Scalp potentials are generated from a mixture of current dipoles distributed throughout the brain (Luck, 2005). It is impossible to know the exact location of a current dipole underlying a given scalp voltage distribution. Volume conduction through inhomogeneous media including neural tissue and the skull further limits spatial resolution. Adding recording electrodes in combination with increasingly sophisticated head modeling approaches and source localization techniques can improve spatial resolution, but regardless, true 3-D spatial localization of current dipoles is not currently possible. A second restriction is the fact that ERPs triggered by external sensory stimuli (e.g., SEPs) depend on ascending pathway integrity. Despite these limitations, the excellent temporal resolution (~1 ms) combined with clinically feasible acquisition procedures make EEG an attractive technique to evaluate changes in brain activity after stroke.

## TMS

TMS uses electromagnetic induction to painlessly elicit electrical eddy currents when held over the skull that can depolarize neuronal membranes and generate action potentials within underlying cortical neuronal tissue (Day et al., 1987; Kobayashi and Pascual-Leone, 2003). Commonly, TMS is delivered over the primary motor cortex (M1) and motor-evoked potentials (MEPs) are recorded by surface electromyography (EMG) of a target muscle. Single-pulse TMS has been used to investigate differences in corticospinal integrity and cortical excitability in stroke (for review: Cortes et al., 2012). Stinear and colleagues have shown that the simple presence or absence of a MEP in the paretic limb can help predict the response to motor skill training in patients with chronic stroke (Stinear et al., 2007) and MEP evaluation is part of a multimodal algorithm used to predict functional outcomes in acute stroke (Stinear et al., 2012). Other TMS assessment techniques are available to evaluate intracortical circuits. Levels of intracortical excitability can be evaluated using paired-pulse TMS (ppTMS) paradigms (Kujirai et al., 1993; Nakamura et al., 1997). Using ppTMS, altered levels of intracortical inhibition and facilitation in both the ipsilesional and contralesional hemispheres in acute and chronic stroke have been demonstrated (Liepert et al., 2000; Manganotti et al., 2002; Bütefisch et al., 2003; Edwards et al., 2013). TMS has also been used to assess interhemispheric interactions in chronic stroke patients (Murase et al., 2004; Takeuchi et al., 2010). Recently, alterations in both transcallosal structure and function were observed in participants with chronic stroke and each associated with upper extremity motor impairment and level of arm motor dysfunction (Mang et al., 2015).

When applied repetitively, (r)TMS can noninvasively and transiently modulate cortical excitability in the human brain (Kobayashi and Pascual-Leone, 2003). Studies have reported that rTMS may be a potential therapeutic adjunct to improve motor performance in patients with acute and chronic stroke (Fregni et al., 2006; Liepert et al., 2007; Conforto et al., 2012). Of particular interest, studies have examined the use of rTMS to restore the balance in cortical excitability between hemispheres by either increasing ipsilesional or decreasing contralesional excitability (Hummel et al., 2008; Hao et al., 2013). Although the neurobiological mechanisms underlying the proposed hemispheric imbalance and the optimal stimulation parameters to modulate excitability in stroke are not well defined, some studies have shown promising therapeutic effects, especially with regards to the treatment of hemi-neglect (Hummel et al., 2005; O’Shea, 2009; Oliveri, 2011). However, substantial inter-individual variability in response to rTMS, unexplained by demographic or lesion characteristics, has kept rTMS applications experimental in stroke.

The dependence on M1 and peripheral pathway integrity in TMS evaluation of stroke neurophysiology limits characterization of neural mechanisms underlying motor dysfunction. Navigated TMS makes it possible to reproducibly stimulate other cortical regions outside M1 but still relies on indirect assessment of cortical excitability using peripheral responses or changes in behavior. Reliance on corticospinal projections for generating MEPs contributes to the inherent variability of MEPs and MEPs may be less reliable in stroke (Butler et al., 2005). In patients with severe stroke where peripheral pathways (e.g., corticospinal tract) may be completely disrupted, it is impossible to generate an MEP and thus characterize cortical excitability limiting insights regarding cortical reorganization in this population of stroke survivors. Furthermore, limited insights regarding connections between M1 and other cortical regions can be gained using MEPs when dual-site paired pulse paradigms are employed (Ferbert et al., 1992; O’Shea et al., 2008).

## TMS-EEG

The strengths and limitations of TMS and EEG in stroke highlight the potential synergistic effects of combining TMS with EEG (Siebner et al., 2009) to create sophisticated paradigms to characterize brain activity by recording evoked potentials from neuronal ensembles at the site of stimulation and activity induced in cortical regions functionally connected to the site (for review: Bortoletto et al., 2015). The ability to transcranially excite a given cortical region to directly record both the local and distributed brain responses removes the reliance on peripheral pathways and behavioral responses. Another advantage of EEG is greater temporal resolution of TMS-related responses compared to other imaging methods such functional magnetic resonance imaging (fMRI). Concurrent TMS-EEG may prove especially useful in patients with damaged peripheral pathways (e.g., patients with severe stroke) by circumventing subcortical structures and directly assessing cortical excitability and connectivity.

**Figure 1:**
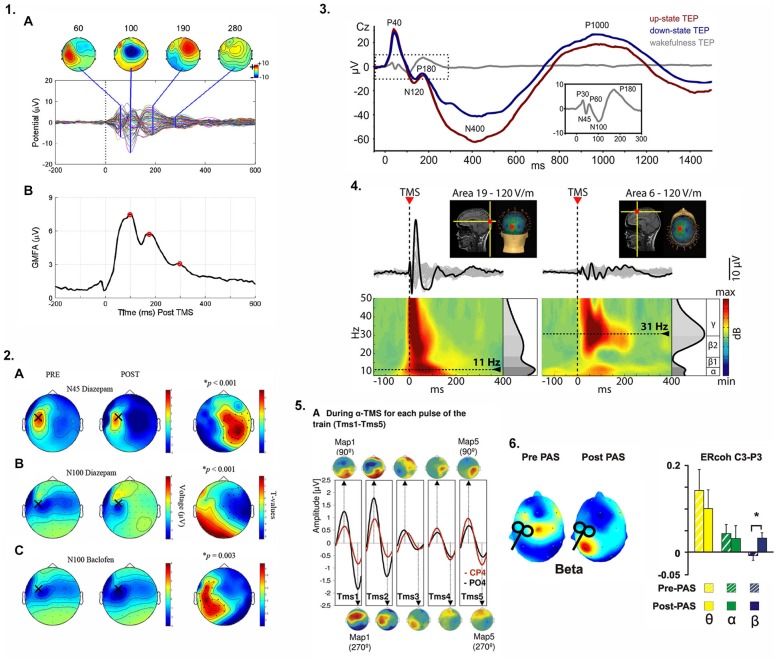
**Examples of approaches previously used to quantify cortical responses to transcranial magnetic stimulation (TMS) and modulate oscillatory activity. (1.1A)**: Spatiotemporal characteristics of TMS-evoked potentials (TEPs) from stimulation over left primary motor cortex (M1), **(B)** Global mean field power (GMFP), a measure of variance across channels, identified periods of maximally accentuated topographical distributions of the TEP to evaluate the global brain responses of TMS over left M1. **(1.2)** Specific components of the TEP waveform are mediated by specific neurotransmitter receptor subtypes. In **(A)** and **(B)** the N45 and N100 components of the TEP are modulated by a gamma-aminobutyric acid (GABA)-A receptor agonist while in **(C)**, the N100 component reflects GABA-B receptor mediated activity (see text and Table 1 for further detail). The topographic plots illustrate the hemispheric distribution of the receptor-specific drug-induced changes in the TEP when stimulating over left M1. **(1.3)** TEPs are modulated by functional brain state. During slow wave sleep (up-state (depolarization) vs. down state (hyperpolarization) of the sleep slow oscillation, red and blue traces respectively), the TEP amplitude not only depends on the current slow oscillation state (phase) at the time of stimulation but the temporo-spatial characteristics of the TEP waveform are completely altered compared to wakefulness (gray trace and inset). **(1.4)** TMS-evoked oscillations reveal site-specific natural frequencies. Occipital stimulation (Area 19) elicited early γ oscillations followed by α oscillations (left) whereas low γ/high β oscillations were observed after frontal cortex (Area 6) stimulation (right). Please note how the time-frequency representation (TFR) of the TEP reflects the temporal aspects of the TEP components, **(1.5)** Repeated TMS pulses delivered at a specific frequency (e.g., α band when stimulating right parietal cortex) entrained naturally occurring local oscillations. With each TMS pulse (TMS1-TMS5), α oscillations within the region of stimulation (CP4 and PO4 electrode locations) were progressively enhanced. **(1.6)** After paired associative stimulation (PAS) of posterior parietal cortex and M1, increased TMS-evoked oscillatory coherence (β only) between two electrodes (C3-P3) near each stimulation site suggests PAS can increase connectivity between stimulated regions in specific frequency bands. Images were reproduced and modified with permission from the following works: **1.1** (Farzan et al., 2013), **1.2** (Premoli et al., 2014a), **1.3** (Bergmann et al., 2012), **1.4** (Rosanova et al., 2009), **1.5** (Thut et al., 2011), **1.6** (Veniero et al., 2013). Refer to original figures for further details and explanation.

Online EEG recording during TMS delivery is methodologically more challenging than offline approaches where EEG is either conducted before or after TMS. Despite the challenges, concurrent TMS-EEG offers the capability to characterize causal connectivity between the stimulated cortical region and other nodes of a network rather than relying on correlative information provided by standalone (e.g., fMRI) or offline imaging approaches (e.g., TMS combined with structural imaging approaches such as diffusion imaging) (Bortoletto et al., 2015). Online TMS-EEG can quantify, interfere with, or modulate neuronal network activity: (i) “Quantification” of network properties relies on the excitation of a certain network node usually by single-pulse TMS and the measurement of its immediate consequences for (oscillatory or evoked) neural activity within that network (“perturb and measure”) (e.g., Rosanova et al., 2009; Figure 1); (ii) “Interference” relates to the disruption of ongoing task-related neural activity by (single-, double-, or burst-) TMS leading to a decrease in task performance (e.g. Capotosto et al., 2012); and (iii) “Modulation” refers to the ability to modify neuronal oscillatory activity, for example via neuronal “entrainment” by means of rhythmic TMS at the frequency of endogenous brain oscillations (e.g., Thut et al., 2011; Figure 1.5). While its main clinical utility may lie in the identification and quantification of biomarkers of chronic stroke, it also appears possible to use TMS-EEG to modulate and thus re-normalize brain activity (Figures 1.5,1.6). For example, TMS-EEG could be used to modulate abnormal beta oscillatory activity during volitional movements (Rossiter et al., 2014) using EEG recordings to guide the timing of TMS delivery over sensorimotor areas in an effort to restore normal patterns of oscillatory activity and potentially improve motor function after stroke.

A characteristic and reproducible EEG waveform (commonly referred to as a TMS-evoked potential or TEP) in response to a single TMS pulse has been repeatedly confirmed (Ilmoniemi et al., 1997; Komssi et al., 2002; Nikulin et al., 2003; Kicić et al., 2008). TEP deflections are time-locked to TMS delivery, presumably representing neuronal activity in both cortical and subcortical regions in healthy individuals. These deflections have been attributed to both fast and slow inhibitory and excitatory post-synaptic potentials evoked by TMS (Ferreri and Rossini, 2013). Importantly, TMS-evoked responses are highly reliable both between participants but even more so within- and between-recording sessions (Lioumis et al., 2009). Thus, unlike MEPs in a muscle, EEG can be used to evaluate cortical responses to perturbation in almost any intra- (Farzan et al., 2013) and inter-hemispheric (Hoppenbrouwers et al., 2010) network of interest, limited merely by the accessibility of the cortical target structure for TMS (i.e., only cortical regions sufficiently close to the scalp can be reached by the magnetic field).

Besides the straightforward analysis of TEP waveforms and their component latencies and amplitudes (resembling typical ERP analysis procedures), several other TEP-based indices have been employed to index certain aspects of cortical reactivity or effective connectivity (Table 1; Figure 1). For example, TMS-evoked oscillations as indexed by their frequency and power and derived from time-frequency representations (TFR) of the average TEP have been shown to emerge in response to a TMS pulse. These TMS-evoked oscillations are specific to the stimulation site (Rosanova et al., 2009) and the current functional brain state (Bergmann et al., 2012). Often, a general index of the brain response to stimulation without further topographical information is the so called “global mean field power” (Skrandies, 1995), which is basically a measure of variance across electrodes and quantifies the topographical diversity of the TEP (thus reaching high values for complex topographies or very local responses) (e.g., Esser et al., 2006). Complex and highly integrative indices have been developed to reflect certain local and network characteristics of the EEG response to TMS (Casali et al., 2010). Another highly interesting index is EEG coherence, a popular measure to study interactions between brain regions and to characterize network connectivity in healthy individuals (Serrien, 2009; Vecchio et al., 2014) and patients with stroke (Wheaton et al., 2008; van Meer et al., 2012). The linear relationship between oscillatory activity in two EEG channels or sources at a specific frequency is measured as coherency and indicates how strongly the phases are coupled to one another. The stronger the coupling, the higher the coherence, interpreted as greater connectivity. This rationale has also been adopted for the measure of inter-trial coherence of TMS-evoked oscillations to study the impact of certain dual-coil rTMS protocols intended to strengthen cortico-cortical effective connectivity (Veniero et al., 2013; Figure 1.6).

**Table 1:** **TMS-evoked potential (TEP)-based indices as possible biomarkers in stroke**.

Index	Quantity indexed	Potential implications for neurophysiology of stroke and stroke recovery
*Multi-channel TEPs (amplitude and waveform)*	Local excitability and spread of activation (see also: **Figures 1.3,1.6**)	Characterize excitability profiles in (non-motor) cortical regions using the summated TMS-related event potential response
*Specific TEP components (amplitude and latency)*	Local intracortical facilitation and inhibition (e.g., N45 and N100 of the M1-TEP for GABA-A- and GABA-B-ergic inhibition) (**Figures 1.2,1.3**)	Assess the integrity of intra-cortical facilitatory and inhibitory circuits
*State-dependency of TEP waveform and components*	Influence of functional brain state on cortical excitability and connectivity (**Figure 1.3**)	Measure influence of functional/arousal state (e.g., during paretic arm movement vs. rest)
*Global mean field power (GMFP)*	Variance of response magnitude across multiple channels (high values for topographically diverse responses) (**Figure 1.1B**)	Estimate of cortical excitability and large-scale network reactivity
*TMS-evoked oscillations (time frequency representation of average TEP)*	Synchronized rhythmic neuronal activity in response to perturbation (**Figures 1.4,1.5**)	Aberrant oscillatory activity (power or frequency) may be a sensitive marker of abnormal information processing. Can be analyzed and manipulated online.
*Interregional coherence of TEP*	Effective connectivity between stimulated and other brain regions (**Figure 1.6**)	Abnormal causal cortical connectivity may identify ineffectual signal propagation in functional brain networks

TMS-EEG has provided candidate biomarkers for a variety of different neurologic conditions. Altered TEPs, in particular a reduced N100 component of the TEP evoked at M1, have been identified in children with attention deficit hyperactivity disorder (ADHD) (Bruckmann et al., 2012; Helfrich et al., 2012), suggesting abnormal intracortical inhibitory activity, likely mediated by GABA-B-ergic pathways (Premoli et al., 2014b). TMS-EEG has been used to assess Alzheimer’s disease severity (Julkunen et al., 2011), and identified a causal link between sleep slow waves, neural plasticity, and cortical information integration (for review: Massimini et al., 2009). Additionally, TMS-EEG-based network connectivity results were able to accurately categorize patients with brain injury across levels of consciousness, suggesting possible therapeutic targets in patients where consciousness is impaired despite preserved cortical connectivity (Sarasso et al., 2014).

TMS-EEG could be used to identify neurophysiologic biomarkers of stroke recovery and prospectively monitor changes in cortical excitability and/or connectivity in response to rehabilitation approaches, pharmacologic therapy, NIBS or spontaneous recovery. For example, TMS-EEG could be used to evaluate changes in sensorimotor cortical connectivity contributing to motor deficits and impairments including hemiparesis, dyscoordination, and spasticity. During paretic arm movement, imbalances in interhemispheric network connectivity between M1 s are considered to be an important contributor to paretic arm dysfunction (Murase et al., 2004). Abnormal interhemispheric connectivity has been measured indirectly, typically while at rest, using techniques unable to elucidate the causal neural mechanisms underlying task-related motor cortical reorganization after stroke (Grefkes and Fink, 2014). TMS-EEG could be applied to address these limitations in an effort to identify the causal contributions of altered motor cortical network connectivity to motor dysfunction after stroke.

The major challenge of the combined TMS-EEG approach is its susceptibility to multiple sources of artifact. When the TMS pulse is administered, several types of artifacts and confounding potentials are recorded in the vicinity of the coil but also more distal sites: (i) Electrical confounds: magneto-electric induction creates fast and high amplitude currents in the EEG electrodes and leads (during stimulation itself and when recharging capacitors) and also electrode-electrolyte interface polarization (Ilmoniemi and Kicić, 2010); (ii) Recording confounds: amplifiers can run into saturation and large signal drifts can occur due to the induced currents; (iii) Mechanical confounds: EEG electrode movements can be caused by due to coil vibration or, more severely, from stimulation of cranial muscles underlying the electrodes (Korhonen et al., 2011); (iv) Biological non-cortical confounds: eye blinks and cranial muscle twitches/potentials can result directly from stimulation or due to a stimulation induced startle response; and (v) Cortical confounds: stimulation of the cutaneous nerves in the scalp and the cranial muscles can trigger somatosensory evoked potentials, and auditory evoked potentials are produced by the audible click of the TMS coil discharge (Daskalakis et al., 2012). Some electrical and recording confounds have been mainly overcome by improvements in stimulator and recording hardware and refinements in experimental techniques. But others such as biological and cortical confounds can only be accounted for by careful experimental design and require additional controls (Ilmoniemi and Kicić, 2010). Signal-space projection (Ilmoniemi and Kicić, 2010), independent component analysis (Korhonen et al., 2011) and advanced subtraction techniques using artifact templates following principal component analysis (Litvak et al., 2007; Levit-Binnun et al., 2010) have been applied to residual artifacts during EEG data processing. Despite the promise of TMS-EEG to elucidate novel contributions of brain activity to behavioral impairment, the approach remains technically challenging which is likely a primary reason it has yet to be applied in stroke.

## Conclusion

Concurrent TMS-EEG has the potential to improve our understanding of the neurobiology of stroke and stroke recovery by offering a sophisticated paradigm to non-invasively characterize human brain excitability and connectivity after stroke. Revealing the causal mechanisms of altered cortical excitability and cortical network reorganization has important clinical implications. If successful, TMS-EEG could provide new tools to improve prognosis, refine treatment approaches, monitor recovery trajectories and individualize care to improve recovery outcomes for patients after stroke.

## Conflict of Interest Statement

The authors declare that the research was conducted in the absence of any commercial or financial relationships that could be construed as a potential conflict of interest.
